# Integrated analysis of the impact of age on genetic and clinical aspects of hepatocellular carcinoma

**DOI:** 10.18632/aging.101531

**Published:** 2018-08-20

**Authors:** Manar Atyah, Yi-Rui Yin, Chen-Hao Zhou, Qiang Zhou, Wan-Yong Chen, Qiong-Zhu Dong, Ning Ren

**Affiliations:** 1 Department of Liver Surgery and Transplantation, Liver Cancer Institute, Zhongshan Hospital, Fudan University, Shanghai 200032, China; 2 Key Laboratory of Carcinogenesis and Cancer Invasion of Ministry of Education, Shanghai 200032, China; 3 Department of Surgery, Minhang Branch, Zhongshan Hospital, Fudan University, Shanghai 201199, China; 4 Institute of Fudan-Minhang Academic Health System, Minhang Hospital, Fudan University, Shanghai 201199, China; 5 Institutes of Biomedical Sciences, Fudan University, Shanghai 200032, China; * Equal contribution

**Keywords:** hepatocellular carcinoma, m RNA expression, gene mutation, copy number variants, AKR1B10, aging

## Abstract

Despite the rapid growing and aging of populations worldwide, our knowledge on hepatocellular carcinoma (HCC) is still age-standardized rather than age-specific, with only few studies exploring the topic from a genetic point of view. Here, we analyze clinical and genetic aspects of HCC in patients of different age groups with the major attention directed to children (≤20 y) and elderly groups (≥80 y). A number of significant differences were found in elderly patients compared to children group, including smaller tumor size (P=0.001) and improved survival rates (P=0.002). Differences in gene mutations, copy number variants, and mRNA expressions were identified between the groups, with alteration rates for some genes like *AKR1B10* increasing significantly with the age of patients. Immunohistochemistry testing of *AKR1B10* showed a significant difference in expression levels at the age of 40 (30.77% high expression rate in patients younger than 40 compared to 51.57% in older patients) (P=0.043). Expression levels also differed between HCC tissues (49.64%) and near-tumor tissues (6.58%) (P<0.001). These findings contribute to the limited data available regarding the age-specific aspects of HCC patients, and support the need to address potential differences in the diagnosis, treatment, and prevention strategies of HCC.

## Introduction

Primary liver cancer is estimated to be the sixth most common cancer worldwide and the second main reason behind cancer mortality. With Hepatocellular Carcinoma (HCC) making up to 80% of its cases, the incidence of liver cancer varies significantly among continents and countries. 75% of all cases are recorded in Asia alone, with China standing for more than 50% of all patients diagnosed [1,2]. Gender, geographic location and ethnicity also affect the incidence rates (with male patients, and Asian populations followed by Hispanics having the highest incidence). Risk factors for HCC also differ among countries. For example, hepatitis B virus (HBV) and aflatoxin B1 (AFB1) are considered as major factors in Asian and African countries [1].

China stands for almost 22% of new cancer cases and 27% of cancer mortality worldwide. The growth and aging of the Chinese population have led to a continuous increase in cancer cases diagnosed [3]. The high incidence of liver cancer in China makes up to 55% of all cases in the world, with HCC ranking as the second major cause in cancer-mortality in males and the third in females. It is the most common type of cancer in Chinese patients under the age of 60 years, with a low incidence before the age of 35 that rises significantly after that age to reach a peak at 80-84 years with higher mortality rates in male patients [3–5].

Since 70% of HBV cases in high incidence areas are infected in perinatal stages or early childhood, the efforts to prevent HBV via vaccination programs in infants and children have shown a huge impact on the incidence rates of HBV (and HBV-related HCC) in targeted age groups. Although vaccinations may be ineffective in almost 5% of cases, still, a decrease of almost 70-85% of HBV-related HCC incidence has been recorded in Asia, and a 95% decrease in HCC related deaths has been recorded in Chinese patients younger than 19 years [1,3].

The success of HCC treatment is associated with early diagnosis. However, in many patients, the diagnosis is only established in advanced stages when a curative treatment is not an available option anymore. Therefore, advanced screening programs have been developed in many Asian countries such as Japan, South Korea, and China. Guidelines suggested that ultrasonography and measurements of blood levels of α-fetoprotein (AFP), AFP-L3, and des-γ-carboxyprothrombin (DCP) can be useful in screening for early stages’ HCC patients [4,6].

In the light of the continuous growing and aging of populations worldwide, and the significant developments of treatment options and disease control strategies, longer life expectancy has been achieved for many diseases, which translated into growing numbers of elderly patients. That created a continuous change in the cut-off of age groups in general and elderly patients in particular. Increased numbers of patients over 70 and 75 years have been recorded, with average life expectancy exceeding 80 years in some countries like South Korea. Therefore, the age cut-off of 80 years for elderly patients group has been accepted and proposed in many reports [2,7 ,8].

The process of aging can be defined as a continuous loss of physiological and biological functions of human organs that increases susceptibility for diseases and injuries. Many theories have been proposed to explain the aging effects on human cells, tissues, and organs. As for the liver, aging can affect both the function and structure of this organ. Changes in metabolic functions, synthesizing proteins abilities, sinusoids and kupffer cells, liver volume and blood flow have already been linked to aging in several reports [8–11].

One theory that has been proposed is that metabolic errors that stand behind the process of aging can actually be caused by both environmental and genetic factors, which may explain the differences among individuals and populations in the susceptibility and the speed of aging [10]. In general, genome alterations (such as DNA damage, mutations, and epi-mutations) are well-known reasons for diseases like cancer; however, they are usually monitored and fixed by the DNA repairing system that preserves the integrity of the genome [12]. Many studies tried to figure out the genetic connection between cancer and the process of aging, considering that the incidence of cancer increases significantly with age. Although cancer is associated with cellular “gain of function“ while aging is defined as a “loss of function“; however, the two have been considered as results of accumulation of cellular damages. Cancers are associated with accumulated mutations in the genome prior to any phenotype changes. Such accumulations have also been linked to aging, considering the time needed for those mutations to happen. However, such a connection has been challenged with findings which suggest that up to 50% of somatic mutations occur in early life of the human body, followed by decreased rate of mutations afterwards. Yet, the changing in tissue context with age can explain the high susceptibility and increased selection for oncogenic cells in advanced age. Another contradiction between aging and cancer is the chromosomal telomeres. The maintenance of telomeres is considered as a hallmark of cancer, while in aged cells short telomeres are a reason for ending the replication of cells. However, studies have found that cancers can indeed arise from short telomeres’ cells, but telomerase activation is still essential for the progression of cancers in the body [11,13–15].

HCC in children is a topic with limited data that requires greater attention and efforts to be explored. Liver cancer is considered to be the third common type of abdominal cancers in children, with HCC ranking second in common liver cancer types (making up to 23% of all cases). However, the high chemoresistance and low cure rates associated with HCC make it an important target for further investigation and research. The majority of pediatric HCC cases occur in patients older than 10 years but the rarity of this disease in childhood makes it very challenging to collect enough cases to carry a wide investigation. Therefore, most of the studies available are retrospective analyses with limited data [16–19].

The majority of pediatric HCC cases lack other background liver diseases such as cirrhosis. Yet, a stronger correlation with HBV infection has been found in childhood HCC, which translated into a significant decrease in incidence following the HBV vaccination programs in infants and children in Asia and the world. That created an urgent need to understand the role of other possible mechanisms of childhood HCC such as mutations and genetic alterations, which are still poorly investigated and addressed [16–22].

Recently, several genes and pathways have been investigated for a potential role in the development of HCC to clarify the genetic background of the disease, one of which is *AKR1B10*. Located at 7q33, *AKR1B10* contains 10 exons and 9 introns, with a length of 13.8 kb and several putative oncogenic and tumor suppressor protein binding sites in its promoter [23]. The detoxification enzyme *AKR1B10* is a well-known aldo-keto reductase family member that has been linked to hepatocellular carcinoma and proposed as a new possible biomarker and an independent risk factor [24–26]. In general, the NAD(P)H-dependent oxidoreductase catalyzes carbonyl compounds and reduces xenobiotic substrates by converting highly reactive aldehydic and ketonic groups into hydroxy groups, which is a form of cell protection against carbonyl toxicity. *AKR1B10* is also linked to retinoid metabolism and fatty acid synthesis [23,24,27]. *AKR1B10* helps in converting retinals to retinols instead of retinoic acids which are considered to be important anti-neoplastic signaling molecules [25,28,29]. Therefore, it is known for its role in intracellular detoxification, cellular proliferation and carcinogenesis [23,30]. Clinically, studies have found that higher levels of *AKR1B10* expression are associated with HBV, HCV, and liver cirrhosis, which may suggest that the expression may be stimulated by the presence of such pre-HCC conditions, with a potential role in early hepatocarcinogenesis [24,26,27]. However, it was also found that high AKR1B10 expression may indicate lower risks of recurrence in HBV-related HCC patients after curative resection [29]. On another note, the relation between *AKR1B10* expression and other clinical aspects like tumor size, lesions number, microvascular invasion, and metastasis is yet to be fully determined [24,29]. Yet, such a relation with tumor grade has been a subject for several studies and a correlation between *AKR1B10* expression and lower tumor stage has been found which may make *AKR1B10* a possible marker for HCC differentiation level [24,28,30]. Several studies have also concentrated on the links between *AKR1B10* expression and drug metabolism, which is extremely important in some therapeutic approaches like chemotherapy. AKRs in general are considered as metabolizing enzymes for carbonyl-containing drugs; therefore, the family of enzymes has been investigated for a potential involvement in chemoresistance [24,28]. Studies showed that *AKR1B10* in specific is linked to drug resistance [31] and its knockdown experiments showed better sensitivity to chemotherapy [28].

Although current studies and researches are devoting greater efforts to uncover more information regarding HCC both clinically and genetically, only few studies are addressing HCC in specific age groups like children and elderly patients, and even fewer studies are exploring this topic from a genetic point of view. Therefore, we, in this study, are shedding more lights on HCC patients by analyzing both their clinical and genetic characteristics in age-specific manner, in order to further investigate the differences among age groups of HCC patients and evaluate the need to address such differences in the diagnosis, treatment, and prevention strategies of HCC.

## RESULTS

### Clinical analysis

Analyzing the clinical variables showed a domination of male patients in both children and elderly groups. However, a higher percentage was recorded in elderly patients. The children group had higher prevalence of HBV positive history than the elderly group, but the sample size of elderly group was much larger. Relevant clinical variables in both groups are summarized in (Table 1).

**Table 1:** A summary of relevant clinical variables in both children and elderly patients that shows the characteristics and differences between the two groups.

Characteristics		Children Group (n=33)	Elderly Group (n=98)	P value
		N (%)	N (%)	
Gender	Male	26 (79)	77 (92)	0.979
	Female	7 (21)	21 (8)	
Age (y)	Range	10~20	80~92	<0.001
	Mean	16.03±2.92	82.49±2.48	
HBV History *	(+)	14 (42.42)	38 (39.17)	0.742
	(-)	19 (57.58)	59 (60.82)	
Over-all Survival (OS) (%)**	(1 Year)	21 (67.74) (n=31)	84 (92.31) (n=91)	0.002
Mortality (%)**	(1 Year)	10 (32.26) (n=31)	7 (7.69) (n=91)	0.002
Tumor Lesions	Single	26 (79)	82 (84)	0.523
	Multiple	7 (21)	16 (16)	
Tumor Size (cm)	Range	1.2~18	1~15	0.001
	Mean	8.73±4.82	5.51±3.12	

* Data for one case in elderly group were lost (n=97).

** Data for some cases were lost, the total numbers of cases available for each group are shown in the table.

**Figure 1:**
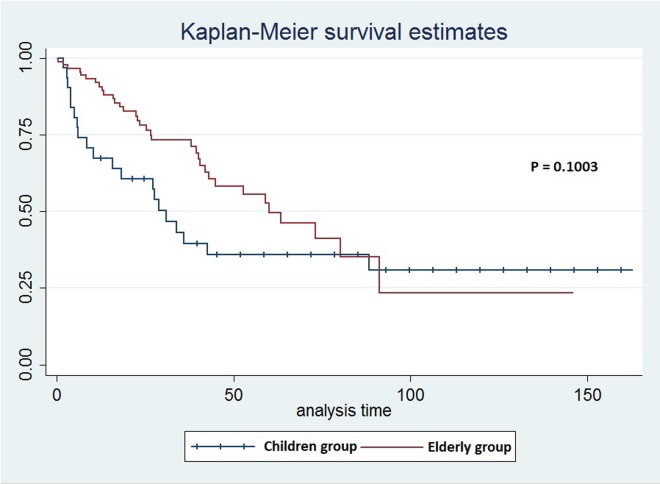
The survival curves for children and elderly groups of patients in the clinical analysis.

The elderly group of patients had better early survival rates (1 year after the surgery) compared to children group. In children group, younger patients (≤15 years old) had decreased survival rates when compared to older children (≥15y) (survival rates were 50% in younger children compared to 76.19% in older children). In the elderly group, survival rates for patients ≤85y were 92.41%, compared to 91.67% in patients ≥86. Survival curves of both groups show that elderly patients have better early survival than children group (Figure 1).

Single lesion tumors were more common than multiple lesions in both groups with a slightly higher rate in elderly patients. However, the average tumor size was bigger in children compared to elderly patients with a difference of more than 3 cm (P=0.001)

As expected, the previous results showed that factors like tumor size are associated with increased risks of mortality in both groups. Tumor size larger than 5 cm was associated with 39.13% mortality rate in children group compared to 12.5% in patients with tumor size ≤ 5 cm (P=0.172). As in elderly group, the difference was statistically significant (P=0.018) with tumors larger than 5 cm being associated with 15.39% mortality rate compared to only 1.93% in ≤ 5 cm tumors. The age influence on the survival of patients with tumors larger than 5 cm was also significant (P=0.036), with elderly patients having a 84.61% 1 year survival rate compared to 60.87% in children.

### Genetic and mRNA alterations

365 patients from TCGA database [32,33] were included in the analysis of genetic alterations, all diagnosed with HCC liver cancer. Male patients were 247 (67.7%) and female patients were 118 (32.3%). All ethnicities were included in the analysis. The children group included 5 patients aged 16 to 20 years, with 2 Asian patients and 3 Caucasians. The elderly group included 14 patients aged 80 to 90 years, with a majority of Caucasian patients. Three aspects of genetic alterations were considered in our study: gene mutations, copy number variants (CNVs), and mRNA expression.

### Genes mutations

Data of all patients in both children and elderly groups were checked for gene mutations. In children group, only three genes (*BIRC6, NRXN1*, and *ZNF676*) were found to be mutated in more than one patient (each gene was mutated in two cases). Due to the presence of mutation patterns in patients, the mutual exclusivity between genes was also investigated and only one pair of genes (*NRXN1/ ZNF676*) showed significant relation (P=0.008). Same method was carried in the elderly group and 14 genes were found to be mutated in at least three patients of this group. The genes are: *ALB, FRAS1, TP53, TTN, CACNA1E, CCDC141, CTNNB1, DNAJC28, KMT2D, LRP2, MSLN, NBAS, RP1*, and *TEP1*. Among those genes, three (*TP53*, *CTNNB1*, and *KMT2D*) were associated with driver mutations. Of which, driver mutations of *TP53* and *CTNNB1* were present simultaneously with mRNA expression alterations. Mutation rates of previous genes in all age groups are shown in (Table 2), while driver mutation rates are shown in (Table 3).

**Table 2:** All age groups mutation rates of the genes mutated in children and elderly groups.

Gene	All Patients (%) (N=365)	Group ≤20 (%) (N=5)	Group 21~40 (%) (N=26)	Group 41~60 (%) (N=145)	Group 61~79 (%) (N=175)	Group 80≤ (%) (N=14)
TP53	30.96	0	30.77	36.55	27.43	28.57
TTN	27.39	0	23.07	22.06	33.14	28.57
CTNNB1	26.57	0	26.92	22.06	31.42	21.42
ALB	11.23	0	7.69	6.89	13.71	35.71
CACNA1E	8.49	0	3.84	4.83	11.43	21.42
FRAS1	6.85	0	7.69	3.45	8	28.57
KMT2D	5.75	20	7.69	4.14	5.14	21.42
BIRC6	5.48	40	7.69	2.76	6.29	7.14
LRP2	4.93	0	3.84	4.14	4.57	21.42
RP1	3.56	0	0	1.38	4.57	21.42
NBAS	3.28	0	3.84	2.07	2.85	21.42
TEP1	3.28	0	3.84	1.38	3.42	21.42
CCDC141	2.46	0	0	2.76	1.14	21.42
NRXN1	2.19	40	3.85	3.44	0	0
ZNF676	1.91	40	0	0	2.86	0
DNAJC28	1.09	0	3.84	0	0	21.42
MSLN	1.09	0	0	0.69	0	21.42

**Table 3:** All age groups driver mutation rates of the three genes found with driver mutations in children and elderly groups.

Gene	All Patients (%) (N=365)	Group ≤20 (%) (N=5)	Group 21~40 (%) (N=26)	Group 41~60 (%) (N=145)	Group 61~79 (%) (N=175)	Group 80≤ (%) (N=14)
CTNNB1	24.65	0	23.07	20	29.71	21.43
TP53	12.6	0	7.69	11.03	14.85	14.28
KMT2D	2.19	0	3.84	1.38	2.28	7.14

### Copy Number Variants (CNVs)

All patients in children group were checked for CNVs and 6 genes were found altered in more than one patient. The genes are *AHCTF1, CNST, KIF26B, SCCPDH, SMYD3,* and *TFB2M*. Alterations included both deletions and amplifications. All six genes shared the same cyto-band (1q44) and were shared by the same two patients with a significant tendency towards co-occurrence among all genes (P<0.001). The alteration rates of the genes in all age groups were 40% for children group (≤20y), 11.54% for (21-40y) group, 7.58% for (41-60y) group, 9.71% for (41-60y) group, and 7.14% for elderly group (≥80y). In the elderly group, 299 genes were altered in three or more patients. Of those genes, 170 were presented in five patients (35.7%), all of which were deletions associated with cyto-bands: 8p21.2 , 8p21.3 , 8p22 , 8p23.1 , 8p23.2 , and 8p23.3. 62 genes were presented in four patients (28.57%), deletions, on cyto-bands: 8p12 , 8p21.1 , 8p21.2, 8p23.1, 8p23.2, and 8p23.3. Genes presented in three patients (21.43%) included 37 amplified genes (associated with cyto-bands: 6p21.1, 6p22.3, 6p24.1, 6p25.2, 6p25.3, and 17q25.3) and 30 deletions (cyto-bands: 6p12.3, 8p11.22, 8p11.23 , 8p12).

### mRNA expression

**Figure 2:**
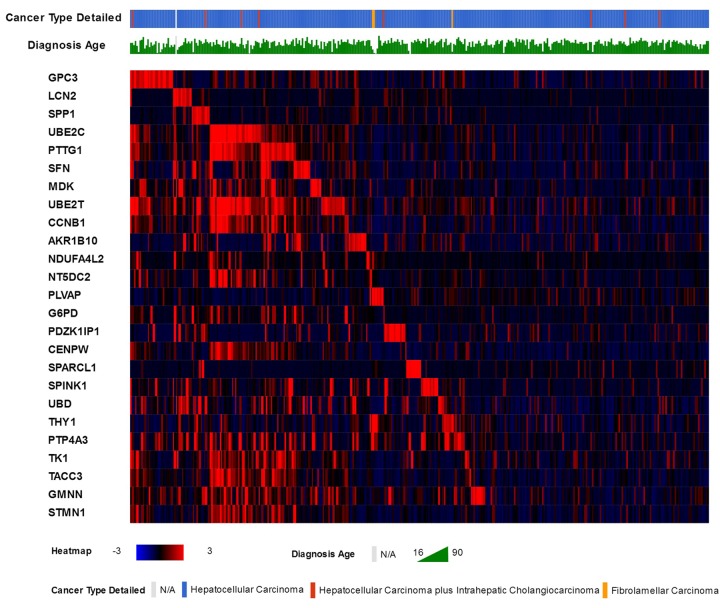
The 25 commonly over-expressed genes in HCC patients and the heat-map of their expression patterns in TCGA liver cancer patients, along with patients liver cancer type and age of diagnosis.

**Figure 3:**
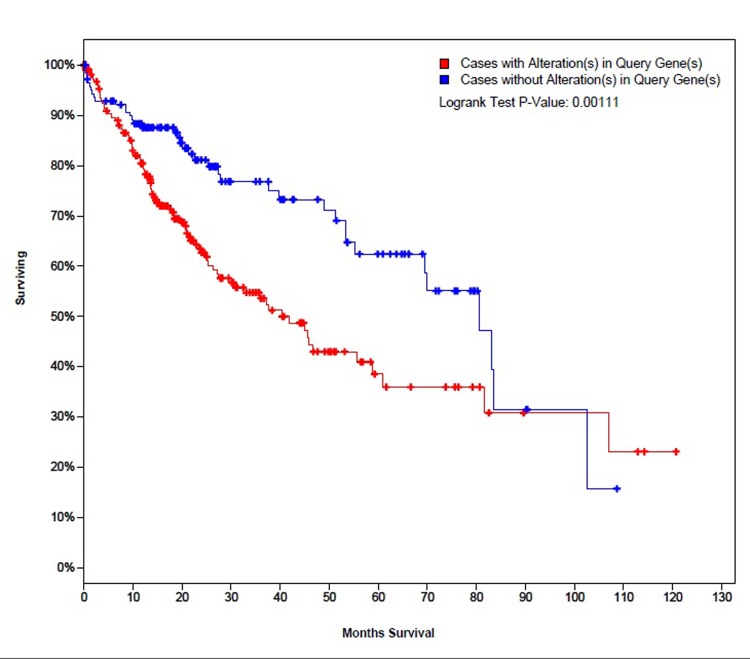
The survival curves of TCGA liver cancer patients with and without alterations in mRNA expression patterns of the selected 25 genes.

25 genes that are commonly over-expressed in HCC patients [34] were selected to be analyzed. The genes are: *GPC3, LCN2, SPP1, UBE2C, PTTG1, SFN, MDK, UBE2T, CCNB1, AKR1B10, NDUFA4L2, NT5DC2, PLVAP, G6PD, PDZK1IP1, CENPW, SPARCL1, SPINK1, UBD, THY1, PTP4A3, TK1, TACC3, GMNN*, and *STMN1*. Expression patterns of all 25 genes in TCGA liver cancer patients along with tumor type and the diagnosis age of patients are shown in (Figure 2) [32,33]. The overall survival (OS) analysis showed that patients with alterations in selected genes expression levels had lower survival rates than those cases without alterations, the differences were statically significant (P=0.0011) (Figure 3) [32,33]. A further investigation of mRNA expression patterns in children group showed that 8 genes were found over-expressed in patients from this group: one patient had both *GPC3* and *UBD* upregulated, *UBE2C* and *UBE2T* in one patient, *PLVAP* and *THY1* in one patient, and *SPARCL1* and *PTP4A3* in one patient. Based on those results, the four pairs of genes were investigated for mutual exclusivity or correlation in expression patterns. Two pairs (*UBE2C* / *UBE2T* and *PLVAP* / *THY1*) were found to have significant tendency towards co-occurrence (P<0.001).

As for the elderly group, 12 genes were over-expressed in patients: *GPC3, LCN2, SFN, MDK, AKR1B10, PLVAP, G6PD, PDZK1IP1, SPINK1, UBD, PTP4A3*, and *TACC3*. *SFN* had the highest rate of alterations in this group (21.42%) followed by *GPC3* and *AKR1B10* (14.28% each). After analyzing the expression patterns in each patient of this group, 4 couples of genes were found to have significant tendency towards co-occurrence: *MDK* / *TACC3*, *SFN* / *G6PD*, *SFN* / *AKR1B10* (P<0.001), and *SFN* / *UBD* (P=0.025). The alteration rates of expression of all 25 genes in all groups are shown in (Table 4). The results showed that *AKR1B10* up-regulation rates increased significantly with the age of patients; therefore, we selected this gene as a subject for a further IHC analysis to investigate the relation between its expression levels and the age of patients.

**Table 4:** The alteration rates of mRNA expression of the selected 25 genes in the five age groups of the genetic analysis.

Gene	All Patients (%) (N=365)	Group ≤20 (%) (N=5)	Group 21~40 (%) (N=26)	Group 41~60 (%) (N=145)	Group 61~79 (%) (N=175)	Group 80≤ (%) (N=14)
UBE2T	16.99	20	23.08	17.93	16.57	0
PTTG1	11.51	0	11.54	12.41	7.43	0
PTP4A3	11.51	20	11.54	13.1	10.28	7.14
UBE2C	9.59	20	11.54	12.41	7.43	0
UBD	9.04	20	7.69	10.34	8.57	7.14
GMNN	8.77	0	15.38	12.41	5.71	0
TK1	7.73	0	7.69	8.96	7.43	0
CCNB1	7.39	0	3.85	7.59	8.57	0
GPC3	7.12	20	15.38	7.59	4.57	14.28
AKR1B10	7.12	0	3.85	5.52	8.57	14.28
PDZK1IP1	7.12	0	0	7.59	8	7.14
SPINK1	7.12	0	3.85	7.59	7.43	7.14
SFN	6.85	0	15.38	5.52	5.71	21.42
MDK	6.85	0	7.69	8.27	5.71	7.14
NT5DC2	6.57	0	7.69	9.65	4.57	0
TACC3	4.93	0	3.85	6.89	3.43	7.14
STMN1	4.93	0	3.85	6.89	4	0
G6PD	4.11	0	3.85	4.83	3.43	7.14
CENPW	4.11	0	7.69	4.14	4	0
NDUFA4L2	3.56	0	7.69	3.45	3.43	0
LCN2	3.29	0	3.85	4.83	1.71	7.14
SPP1	3.29	0	3.85	2.76	4	0
SPARCL1	3.01	20	3.85	1.38	4	0
THY1	3.01	20	0	3.45	2.86	0
PLVAP	1.64	20	3.85	0.69	1.14	7.14

### AKR1B10 expression

**Figure 4:**
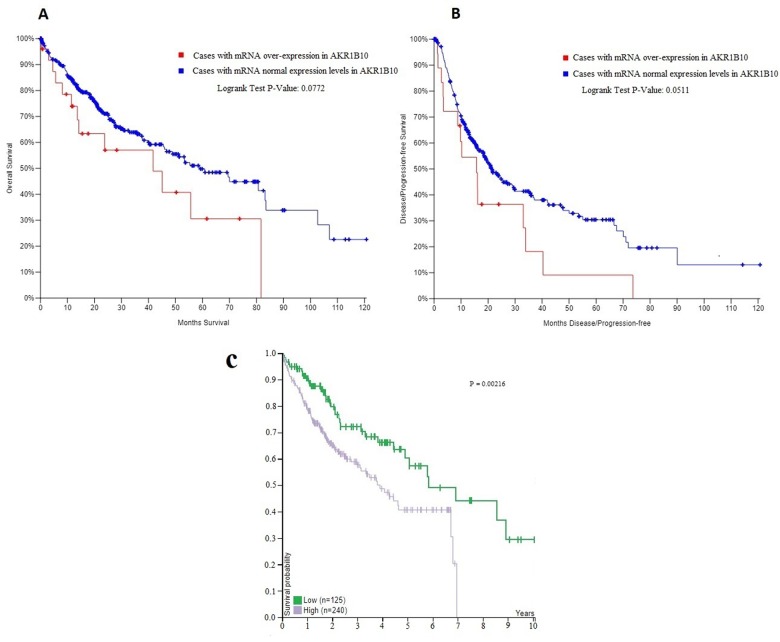
(**A**, **B**) The overall survival (OS) and disease free survival (DFS) curves in patients with and without mRNA over-expression in AKR1B10. (**C**) The correlation between AKR1B10 mRNA expression level in liver cancer and patient survival from the pathology atlas of the human cancer transcriptome .Corresponding expression cutoff= 26.8 FPKM. 5-year survival for patients with high expression= 41% , 5-year survival for patients with low expression= 57%, and log-rank P value = 0.00216. URL: https://www.proteinatlas.org/ENSG00000198074-AKR1B10/pathology/tissue/liver+cancer#ihc.

**Figure 5:**
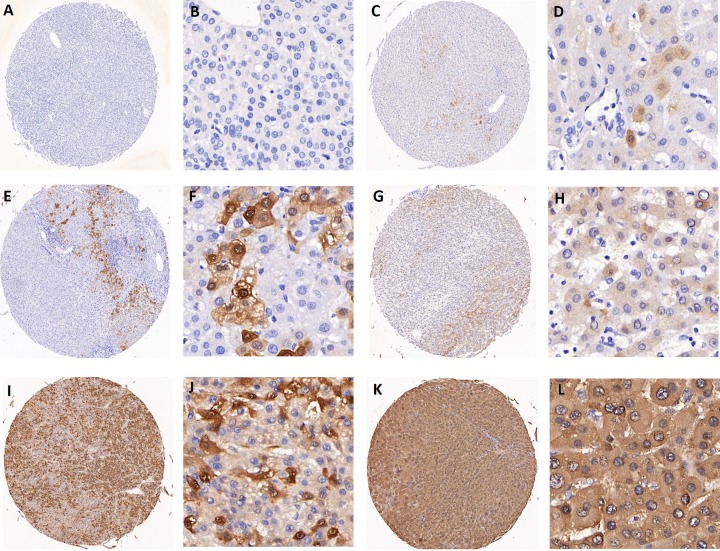
Representative images of AKR1B10 immunohistochemistry staining in tissues. (**A**-**B**) represent negative staining (score: 0). (**C**-**D**) (score: 1), (**E**-**F**) (score: 2), (**G**-**H**) (score: 4) all represent weak staining. (**I**-**J**) (score: 6) and (**K**-**L**) (score: 8) represent strong staining. Scores 0 to 4 are categorized as low expression of AKR1B10, while scores 6 to 8 are considered as high expression.

According to TCGA database and the pathology atlas of the human cancer transcriptome, the overall survival (OS) and disease free survival (DFS) analyses showed that patients with normal *AKR1B10* expression levels have better survival rates than those with over-expression (Figure 4) [32,33,35]. Therefore, and based on our previous results, we chose to carry a further IHC analysis of *AKR1B10* to investigate a potential relationship between its expression levels and the age of patients. Representative images of *AKR1B10* immunohistochemistry staining in tested HCC tissues are provided in (Figure 5). Samples from 280 patients (280 cancer tissue samples and 168 near-tumor samples) were collected and tested. All relevant clinical data are summarized in Table 5.

**Table 5:** A summary of relevant clinical data of 280 patients in AKR1B10 IHC expression analysis.

**N (%) (280)**	**Low expression** **141 (50.36)**	**High expression** **139 (49.64)**	**P**
GENDER (M/F)	120/21	123/16	0.403
HBsAg (+/-)	117/24	116/23	0.915
Cirrhosis (+/-)	85/56	85/54	0.882
MVI * (181) (+/-)	41/50	36/54	0.492
BCLC (0+A/B+C)	53/88	60/79	0.342
Tumor Differentiation * (278) (I+II/III+IV)	89/50	97/42	0.308
AFP (Normal/ Elevated)	47/94	58/81	0.147
CEA * (181) (Normal/ Elevated)	84/7	82/8	0.770
CA19-9 * (181) (Normal/ Elevated)	68/23	71/19	0.507
Number of lesions (single/multiple)	117/24	108/31	0.266
Tumor Size (<=5/>5)	74/67	80/59	0.394
Encapsulation (yes/no)	78/63	78/61	0.893
Tumor thrombus (yes/no)	26/115	32/107	0.344
ALT (Normal/ Elevated)	90/51	79/60	0.232
AST * (181) (Normal/ Elevated)	62/29	57/33	0.496
ALP * (181) (Normal/ Elevated)	62/29	64/26	0.663
GGT (Normal/ Elevated)	61/80	45/94	0.06
TB * (273) (Normal/ Elevated)	123/16	119/15	0.934
DB * (174) (Normal/ Elevated)	82/7	77/8	0.716
ALB * (271) (Normal/ Elevated)	8/130	9/124	0.742

* represents the total number of patients data are available for.

**Figure 6:**
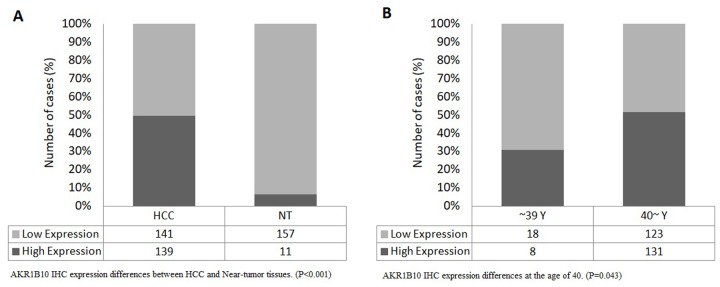
(**A**) AKR1B10 IHC expression differences between HCC and Near-tumor tissues. (P<0.001), (**B**) AKR1B10 IHC expression differences at the age of 40. (P=0.043).

The results showed statistically significant differences between expression levels in HCC tissues and near-tumor tissues (P<0.001), with a high expression rate of 49.64% in HCC samples compared to only 6.58% in near-tumor samples (Figure 6A). We also found that expression levels differed significantly (P=0.043) at the age of 40 (Figure 6B), with a rate of 30.77% of high expression in patients younger than 40 compared to 51.57% in older patients.

## DISCUSSION

Our knowledge regarding HCC has been continuously expanding. Currently, more studies have been addressing new aspects such as HCC genetic background and novel treatments. Many genes have been explored and linked to a certain role in the development or treatments of HCC. However, only few efforts are dedicated to understand the differences and characteristics of HCC in certain groups like elderly patients or children, and even fewer studies are exploring the genetic features specific to them. Therefore, we carried, in this study, a clinical and genetic (gene mutations, CNVs, mRNA expression) analysis of elderly and young HCC patients, in order to contribute to the limited knowledge regarding this topic and help provide a solid base for further investigations.

Considering the growth and aging of populations all over the world (especially in China) and the rarity of HCC in certain age groups like children, we chose the age of 20 and 80 years as cut-off points for the young and elderly groups of patients.

In the clinical analysis, the sample size of the elderly group was larger than the children group due to the rarity of HCC in young patients after the national program of HBV vaccination in infants and children [17]. The majority of patients in both groups were males with a higher percentage in elderly group (92%) compared to (79%) in young patients. A possible explanation could be that several risk factors for HCC (like alcohol consuming and chronic HBV history) are more common in older males [9].

In general, the process of aging and the changes it brings can explain a lot of differences among elderly patients and other age groups. The accumulation of molecular abnormalities with time, the age-related alteration in immune functions and other changes have already been pointed out for their association with cancer morbidity and mortality [36]. However, when compared to children group, the elderly group in our study showed higher rates of early survival. The better liver functions in elderly patients and the heavier burden of the tumor on young livers can lead to such differences [8]. Besides, the tumor is unlikely to grow too large without leading to organ failure or death in earlier ages [9]. The differences between age subgroups within each group also indicated the impact of age on survival of patients, with older children and younger elderly having better survival rates than other patients in their groups. However, the effect of aging itself on survival rates and the role of other causes of death in elderly group prevent us from drawing a solid conclusion, and a further investigation with better controlled data is advised.

As expected, our results showed that tumor size and lesions number can affect the survival in both groups. The majority in both groups was presented with single lesions. The average tumor size, however, was bigger in children which translated into increased risk of death; an increase that can be expected considering the smaller size of liver in younger patients.

The number of studies addressing the genetic aspect of HCC is rapidly growing, expanding our knowledge of related genes and their role in diagnosis and treatment. However, the majority of studies are age-standardized rather than age-specific, leaving doctors and care givers with an only choice of applying the finding of those studies in special age groups like children and ignoring the potential differences related to the age of patients. In children, the family history can be taken into consideration; however, for De novo alterations such a history is not useful. Therefore, doctors tend to check for common alterations, considering the lack of age-specific data. As for elderly patients, aging has been investigated for its role in cancer development in general (such as the accumulation of alterations and the compromising of DNA repair system) [12,13]. However, the current knowledge in this field is still falling behind when compared to age-standardized data, and further efforts are required to optimize the diagnosis and treatment plans. Therefore, we carried an age-specific genetic analysis that covers three main aspects: gene mutations, copy number variants (CNVs), and mRNA expression patterns.

The analysis of gene mutation patterns in children group showed that only three genes were mutated in more than one patient (*BIRC6*, *NRXN1*, and *ZNF676*), with *NRXN1* and *ZNF676* showing a significant tendency towards co-occurrence. Although such findings could indicate a role of certain genes in young HCC patients; however, the limited number of patients in this group makes it hard to draw a conclusion, and further research is recommended. The protein encoded by *BIRC6* is known for its role in inhibiting cell’s apoptosis, while proteins encoded by *NRXN1* acts as adhesion molecules in cells of the nervous system [37–39]. For the elderly group, 14 genes were mutated in more than three cases (21.42%). *ALB* had the highest rate with (35.71%). The majority of genes were presented with passenger mutations except for *TP53*, *CTNNB1*, and *KMT2D* having driver mutations. Of which, *TP53* and *CTNNB1* mutations were associated with mRNA expression irregularities, which emphasize their role in cancer development. Mutations in these three genes have already been observed in different types of cancers [37–39]. As for *TP53*, P53 pathway is known for its association with aging and diseases development in advanced ages since P53 functions include suppressing tumors, preserving genome integrity, and restricting the proliferation of damages cells [40]. Genes like *CCDC141*, *DNAJC28*, and *MSLN* showed higher prevalence in elderly patients than other age groups, while the incidence of mutations in genes like *CACNA1E*, *LRP2*, and *RP1* increased with the age of patients. Those mutation patterns show that the process of aging plays a different role in different age groups, with those genes being a potential key to uncover such a role. Therefore, searching for age-specific alterations is vital to optimize the care we give to patients of specific ages, like elderly patients.

Copy number variants (CNVs) of DNA sections can overlap with certain genes and, therefore, alter their functions, leading to diseases like cancer [12]. Six genes were found to be altered in more than one patient in children group. The significant tendency of co-occurrence among all six genes (P<0.001) led us to investigate the location of those genes on human chromosomes. All six genes shared the same cyto-band: 1q44; therefore, we suggest this location to be further investigated for a relation with HCC in all patients in general (since 9.31% of all patients had an alteration in this cyto-band) and children in specific. 1q44 has been linked in other studies to abnormalities like microcephaly, seizures, and other diseases [41]. More genes were found in the elderly group (299 genes). The majority of them were located in chromosome 6 and 8 in cyto-bands like: 6p21-25, 8p11-12, and 8p21-23. Abnormalities in some of those locations like 8p21-23 have already been associated with several cancers like lung, breast, colon, brain, prostate, and even liver cancer [42], and the deletion of 8p has been proposed as a prognostic predictor in HCC patients [43]. The importance of such findings is that it gets us closer to be able to achieve a diagnosis based not only on phenotypic or genetic changes but also karyotypical changes, which may save both efforts and resources.

Out of 25 genes that were considered for mRNA expression analysis, 8 were up-regulated in children group. Among those genes, three (*PLVAP*, *SPARCL1*, and *THY1*) were associated with low rates of up-regulation in other age groups. Therefore, a primary consideration of those genes in age-specific diagnosis is highly advised for further investigation. *THY1* gene encodes a protein that is associated with cell adhesion and communication. The gene is also involved in tumor suppressing, metastasis, and prognosis of many cancer types like gallbladder and nasopharyngeal carcinoma [37–39,44]. *PLVAP* has also been investigated as a therapeutic target in HCC [45]. mRNA analysis also showed a tendency towards co-occurrence in two pairs of genes: (*UBE2C* / *UBE2T* and *PLVAP* / *THY1*). A further investigation of the mechanisms and the roles of the given genes could help uncover age-specific patterns of alterations. For example, both *UBE2C* and *UBE2T* encode ubiquitin-conjugating enzymes that are needed in ubiquitination and cell cycle progression [37–39]. The genes have already been linked to several types of malignancies such as bladder cancer, malignant glioma, and Fanconi anemia [46–49].

12 genes were up-regulated in elderly patients, with *SFN, GPC3, AKR1B10* having the highest rates (21.42%, 14.28%, 14.28%, respectively). Comparing this group’s rates to other age groups’, we found that up-regulated patterns for genes like *PLVAP* and *AKR1B10* are more common in elderly patients, which may indicate a certain relation with advanced age. The same could be considered for genes like *PDZK1IP1* that was found to be up-regulated only in patients older than 40 years. Out of the 12 genes selected in elderly group, *AKR1B10* stood out as its up-regulation rates increased significantly with the age of patients. As mentioned earlier, this gene has already been linked to cell proliferation and differentiation and addressed in HCC and other types of tumors like lung and breast cancer [49]. Among the four pairs of genes found to be significantly correlated, (*SFN / G6PD*) and (*SFN / AKR1B10*) could be of a certain use in future age-specific approaches, considering their high prevalence in elderly patients and stronger relation to aging.

Based on the previous findings, we selected (*AKR1B10*) as a subject for a further immunohistochemistry (IHC) testing in order to analyze the influence of its expression levels in HCC. Our results showed statistically significant differences between *AKR1B10* expression levels in HCC tissues and near-tumor tissues (P<0.001), with a high expression rate of 49.64% in HCC samples compared to only 6.58% in near-tumor samples. Those findings are in line with other studies in which high expression levels of *AKR1B10* have been linked to HCC while expression levels in near-tumor tissues, normal tissues, and even benign liver lesions showed minimal to no expression levels of the gene, which makes *AKR1B10* a potential biomarker for the diagnosis of HCC [27,28,50].

Another finding of our study is that *AKR1B10* expression levels showed a significant difference at the age of 40 (P=0.043), with a high expression rate of 30.77% in patients younger than 40 compared to 51.57% in older patients. These results validate and emphasize the proposed relationship between *AKR1B10* expression and the age of patients. Due to the rarity of HCC in patients of certain ages (especially younger than 20 years old), an independent comparison of patients younger than 20y and older than 80y was hard to be carried.

**Figure 7:**
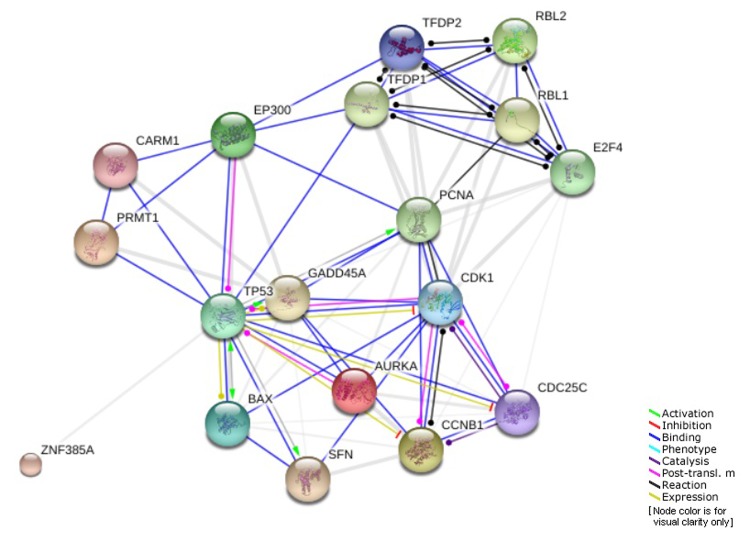
Genes involved in G2 cell cycle arrest pathway regulated by TP53.

As we mentioned earlier, the promoter region of *AKR1B10* includes several oncogenic and tumor suppressor protein binding sites, one of which is *TP53* tumor suppressor [23,24,29]. Based on the previous results, the percentage of *TP53* driver mutations was much higher after the age of 40. In addition, *AKR1B10* was found to be significantly correlated with *SFN* regarding mRNA expression levels with high prevalence in elderly patients. *SFN* is one of the genes involved in G2 cell cycle arrest pathway that is regulated by *TP53*. The genes involved in this pathway and their interactions are shown in (Figure 7) [51]. All of that suggest a potential involvement of this specific pathway and other similar ones in the role *AKR1B10* plays in different age groups of HCC patients.

The transcription factor nuclear factor erythroid 2-related factor (Nrf2) is known for its involvement in the regulation of *AKR1B10* in addition to its role in the adoptive response to oxidative stress and damage [25,52], which is considered as one leading theory of aging [53]. Oxidative stress refers to the accumulation of reactive oxygen species (ROS) and reactive nitrogen species (RNS) in cells, exceeding the cellular antioxidant capacity, and therefore, damaging cellular DNA, lipids, and proteins [54,55]. Levels of accumulation increase with age, leading to a significant decline in cellular functions and repairing systems (including DNA repairing system) [53]. In general, hepatic cells have a high antioxidant ability when compared to other types of human cells; however, due to the functions of hepatic cells, accumulation of oxidative damage is quite easy to happen [56]. A study has shown that, with age, hepatic cells show a significant increase in protein carbonylation, lipid perioxidation, in addition to a decrease in antioxidant enzymes activities [57], which may easily explain how genes with a mechanism like *AKR1B10* and their high expression can be involved in the increase of carcinogenesis with age.

The effect of genetic alterations and profile on the phenotype or clinical aspects of cancer patients is very important. However, genetic alterations or signatures in general can fall into three groups: Predictive, functional, or phenotypical alterations [58,59]. Therefore, passenger mutations can also be extremely useful for early screening and diagnosis of HCC which could enhance the success of treatments offered. We still encourage future studies and researches to explore possible relations between the genotype and phenotype of HCC patients in better controlled study designs.

Like all other researches in this field, we were faced with certain limitations in this study. The limited number of available data and patients (especially in children group) made it hard to draw definite conclusions based on the results. It also decreased our ability to control the conditions of the analysis to the level we wanted or find patients with certain level of similarity. The rarity of this disease in some age groups forced us to carry the analysis retrospectively. In addition, we decided to include patients from all ethnicities in the genetic analysis, considering the lack of relevant data. Such limitations are often encountered in studies like ours, which sometimes may translate into different results among different studies. However, we still believe that the contribution of this study to the limited data in this field and the results we found (especially the genetic findings) can indeed create a strong base for future researches and studies to uncover more age-specific abnormalities and carry deeper analyses in targeted age groups. Hopefully, similar future studies can optimize the diagnosis and treatment plans offered to young and elderly HCC patients in both Asian and global populations.

## MATERIALS AND METHODS

### Patients

Different groups of patients were selected for each part of this study:

For the clinical analysis, data from Department of Liver Surgery, Zhongshan Hospital, Fudan University were reviewed for the selection of suitable patients. From 2000 to 2015, a total of 131 patients’ data were selected to be retrospectively analyzed. All patients were i) ≤20 or ≥80 years old (in order to include sufficient number of patients, considering the rarity of HCC in younger populations and the increased numbers of HCC patients older than 80y in Asia and China [2,7,8], ii) pathologically diagnosed with HCC liver cancer, and iii) underwent hepatectomy in Zhongshan Hospital, Fudan University. Patients were then divided into two groups according to their age at the time of surgery: children group (20 years old or younger, 33 patients) and elderly group (80 years old or older, 98 patients). Relevant clinical variables and information including age, gender, HBV status, tumor size, lesions number, and date of surgery were collected and analyzed. Survival periods of patients were calculated starting from the date of surgery according to follow-up results in 2016. The study was approved by the Ethics Committee of Zhongshan Hospital, Fudan University and carried in accordance with the Helsinki declaration.

For genetic characteristics, a total of 365 HCC patients (from all age groups and ethnicities) were selected from TCGA database to be genetically analyzed and compared, with the major attention directed to two groups in specific: childhood patients (≤20y, n=5) and elderly patients (≥80,n=14). Other age groups include: (21-40y,n=26), (41-60y,n=145), and (61-79y, n=175). The genetic analysis included three different aspects: genes mutations, copy number variants (CNV), and mRNA expression levels.

For the further investigation of *AKR1B10*, data and tissue samples from 280 patients (280 cancer tissue samples and 168 near-tumor samples) were collected from Department of Liver Surgery, Zhongshan Hospital, Fudan University. All patients were i) pathologically diagnosed with HCC liver cancer ii) underwent hepatectomy in our hospital between 2005 -2010. Immunohistochemistry was then performed and samples were divided into high expression and low expression groups.

### Genetic and mRNA alterations

To address the limitations and lack of genetic data on HCC childhood and elderly patients worldwide, we based the genetic analysis in our study on TCGA global database for liver cancer. Platforms like cBioPortal (http://www.cbioportal.org) and UALCAN (http://ualcan.path.uab.edu/index.html) were used to collect and analyze relevant data. cBioPortal is a platform that helps download, analyze and visualize genetic data from large scale cancer databases like TCGA database [32,33]. UALCAN is another platform that provides gene expression information and survival analyses regarding different types of tumors [34].

Alterations from three different aspects were considered: genes mutations, copy number variants (CNV), and mRNA expression levels.

For genes mutations analysis, mutated genes in childhood and elderly groups were analyzed. Mutated genes shared by two or more patients in childhood patients group, or by three or more patients in elderly patients group were selected for further investigation. Mutation rates of selected genes were calculated in all age groups and the mutual exclusivity were determined. Driver mutations were then selected and analyzed to figure any possible connections to mRNA expression alterations.

For copy number variants (CNVs), CNVs shared among two or more patients in childhood patients group, or three or more patients in elderly patients group were selected. The CNVs were then assigned to their locations on human chromosomes and analyzed for insights on possible age-related variants.

As for the mRNA expression, 25 commonly over-expressed genes in HCC patients [34] were selected to be investigated. The expression patterns were then analyzed in all 365 patients and the effects of expression patterns on overall survivals (OS) were determined and visualized. Alteration rates in different age groups were then compared, to understand the age influence on expression patterns. Patients in childhood and elderly groups were individually analyzed and genes mutual over-expression exclusivity were determined to spot any age-relevant significant connections that can aid in identifying potential genes in the given age groups.

### Immunohistochemistry (IHC)

Tissue samples were paraffin embedded; therefore, deparaffinization and then rehydration of samples in descending alcohol (ethanol) gradient were carried out first. Peroxidase activities were then blocked by using 3% hydrogen peroxide for 10 min. Samples were placed in 0.1 M citrate buffer and heated for 8 min in a microwave to induce antigen epitope retrieval. After that, 10% goat serum was used before the incubation of samples with primary antibody overnight at 4°C temperature, followed by secondary antibody for 30 min at room temperature. Finally, staining of samples was completed by using 3,3'-diaminobenzidineto visualize the results.

Staining results were assessed by two different doctors separately. The extent of staining was graded on a scale from 0 to 4 (0%, 1~5%, 6~25%, 26~75%, 76~100%) while a scale from 0 to 2 was used for intensity (0: negative, 1: weak, 2: strong). Final scores (0 to 8) were calculated (extent × intensity) and divided into two groups: low level of expression (0~4) and high level of expression (6~8).

### Statistical analysis

Statistical analysis was performed with STATA software (13.0, College Station, Texas 77845 USA) and SPSS software (21.0, IBM corp.). Variables were presented as means ±standard deviations (SD) (for continuous variables) or frequencies (%) (for categorical variables). Overall survival (OS) rates were analyzed in accordance with Kaplan-Meier’s method and the log-rank test. P values were considered statistically significant when <0.05.
